# Deadly Attraction – Attentional Bias toward Preferred Cigarette Brand in Smokers

**DOI:** 10.3389/fpsyg.2017.01365

**Published:** 2017-08-11

**Authors:** Ewa Domaradzka, Maksymilian Bielecki

**Affiliations:** ^1^Institute of Psychology, Polish Academy of Sciences Warsaw, Poland; ^2^Faculty of Psychology, SWPS University of Social Sciences and Humanities Warsaw, Poland

**Keywords:** smoking, attentional bias, dot probe task, eye-tracking, brand preference

## Abstract

Numerous studies have shown that biases in visual attention might be evoked by affective and personally relevant stimuli, for example addiction-related objects. Despite the fact that addiction is often linked to specific products and systematic purchase behaviors, no studies focused directly on the existence of bias evoked by brands. Smokers are characterized by high levels of brand loyalty and everyday contact with cigarette packaging. Using the incentive-salience mechanism as a theoretical framework, we hypothesized that this group might exhibit a bias toward the preferred cigarette brand. In our study, a group of smokers (*N* = 40) performed a dot probe task while their eye movements were recorded. In every trial a pair of pictures was presented – each of them showed a single cigarette pack. The visual properties of stimuli were carefully controlled, so branding information was the key factor affecting subjects’ reactions. For each participant, we compared gaze behavior related to the preferred vs. other brands. The analyses revealed no attentional bias in the early, orienting phase of the stimulus processing and strong differences in maintenance and disengagement. Participants spent more time looking at the preferred cigarettes and saccades starting at the preferred brand location had longer latencies. In sum, our data shows that attentional bias toward brands might be found in situations not involving choice or decision making. These results provide important insights into the mechanisms of formation and maintenance of attentional biases to stimuli of personal relevance and might serve as a first step toward developing new attitude measurement techniques.

## Introduction

Even those of us who are not disco music enthusiasts might understand the logic of Gloria Gaynor’s confession: “You are just too good to be true/Can’t take my eyes off you.” We naturally interpret a prolonged gaze as a sign of interest. Certainly, the relationship between preferences and visual attention is not limited to human faces. We tend to look longer at many other categories of objects we find attractive or choose – both in a lab setting (Pieters and Warlop, 1999) and natural environment (Gidlöf et al., 2013). Gaze length not only reveals but, in some conditions, causally influences our choices as shown by Shimojo et al. (2003). Still, surprisingly little is known about the exact boundaries of the gaze bias effect despite its importance both for basic (Schotter et al., 2010) and applied research (Wedel and Pieters, 2008).

In particular, studies focusing on gaze bias related to processing information about brands and packaging are significantly limited in their scope. Most of them focus directly on the situations of choice or judgment (Pieters et al., 1997; Pieters and Warlop, 1999; Clement, 2007; Clement et al., 2013) and ignore a more basic question: is it possible to detect a “pure” attentional bias related to a particular brand in the context not requiring any decisions. This very question was the starting point of the present paper. The purpose of our research was twofold: firstly, to determine the existence of a gaze bias toward packaging of the preferred brand of cigarettes in smokers and secondly, to investigate which (if any) stages of the stimulus processing are affected by this bias. According to our knowledge, this is the very first study addressing directly both these issues.

Over the last four decades, studies on attentional biases to various types of stimuli have gained much interest. At first, they concentrated on attentional biases evoked by evolutionarily significant stimuli, such as threatening faces, spiders, and snakes (Hansen and Hansen, 1988; Öhman and Mineka, 2001; Öhman et al., 2001; Blanchette, 2006). Further studies revealed that attentional biases might also be related to the emotional state, and therefore individual differences in attentional processing became a topic of interest. For example, elevated level of anxiety relates to enhanced vigilance toward threat-related stimuli (MacLeod et al., 1986; Mogg et al., 2000; Juth et al., 2005; Fajkowska and Krejtz, 2007; Matsumoto, 2010) and problems with disengagement from them (Fox et al., 2001, 2002; Georgiou et al., 2005; Schofield et al., 2012; Leleu et al., 2014). Similarly, depression was linked to prolonged maintenance of attention on sadness-related stimuli (Bradley et al., 1997; Gotlib et al., 2004; Caseras et al., 2007) and avoidance of positive material (Sears et al., 2011). These findings clearly show that the relevance of stimuli plays an essential role in shaping our attentional processing patterns.

It is important to notice, however, that congruence between the stimuli and the viewer’s emotional state is not the only setting in which gaze bias might be observed. Our needs, desires, and motivation might also significantly influence the distribution of visual attention (Theeuwes and Belopolsky, 2012), leading to the preferential processing of stimuli representing reward such as food in studies on hungry (Mogg et al., 1998) or obese participants (Werthmann et al., 2011). Analogous effects were also revealed in users of psychoactive substances including alcohol (Townshend and Duka, 2001; Weafer and Fillmore, 2012), nicotine (Mogg et al., 2003; Field et al., 2004; Kwak et al., 2007), caffeine (Yeomans et al., 2005), and cocaine (Marks et al., 2014).

Given the breadth of research on stimuli associated with reward, we found it surprising that, to our knowledge, none of them focused on detecting attentional bias evoked by specific brands. One of the core functions of branding is to allow effective visual discrimination of products (Zaichkowsky, 2010). The act of product purchase is in many cases habitual, therefore processing of brand visual identification is highly optimized. Brand elements form an implicit representation, and their accessibility is correlated with both brand equity and brand usage (Friedman and Leclercq, 2015). At the same time, buying a product is a highly rewarding activity – a property that, in extreme cases, might even lead to the development of compulsive behavior (Racine et al., 2014). Both these aspects should contribute to the emergence of bias effect among loyal consumers. The existence of such effects is also a logical extension of the more general theories of attentional prioritization emphasizing the close interplay between cognitive and emotional aspects of information processing (Pessoa, 2008). The currently prevailing view of the attentional bias underlines the notion of relevance (Purkis et al., 2011) and, with regard to addiction-related stimuli, more specific mechanism of incentive-salience attribution (Robinson and Berridge, 2000, 2001). This mechanism transforms the neural and psychological representations of stimuli, which leads to perceiving them as attractive, wanted, and – what is of particular interest for us – attention-grabbing.

Because of the exploratory nature of the present research, we decided to study attentional bias toward cigarette brands, as smokers are a group in which, according to the theoretical premises presented above, the chances of detecting the bias are maximized. Smokers are characterized by a very high brand loyalty (Kann, 2012; Cowie et al., 2014) and – due to the nature of the addiction – have optimal conditions to develop an attentional bias. Smokers tend to have their favorite brand of cigarettes which they buy regularly and have everyday, frequent contact with the branded packaging. Moreover, the brand preference is strongly related to the reward expectancy which is postulated to be one of the key mechanisms driving the attentional bias (Field et al., 2011; Jones et al., 2012). As mentioned above, the incentive-related stimuli may cause an increase in reward-seeking motivation (Berridge et al., 2009) which, on the attentional level, should lead to preferential processing of brand-related stimuli.

Before we move on to discuss the details of our study, it is important to notice that although attentional bias toward several categories of personally relevant stimuli is a well-established phenomenon, it is not ubiquitous. For example, some research using sadness-related stimuli failed to replicate the effect in depressed participants (Mogg et al., 2000; Karparova et al., 2005; Sears et al., 2010). Similarly, the enhanced orienting and impaired disengagement evoked by threat expressions in anxious groups has not always been found (Rinck et al., 2003; Derakshan and Koster, 2010). Also, some studies on alcohol users do not fully confirm the expectations based on the personal relevance of stimuli (Fridrici et al., 2012; Christiansen et al., 2015). There are many factors that might contribute to the emergence of these inconsistencies: varying effect sizes, limited power, as well as important differences in the methods being used (types of tasks, stimuli, timing details, etc.). From a theoretical perspective, the most important distinction stems from the fact that attentional bias might be present at different stages of stimulus processing. As already mentioned above, threat processing in anxious individuals is mostly expressed in orienting and disengagement, while in depressed individuals sadness-related material evokes effects reflected in maintenance. Because of the lack of existing data concerning attentional biases evoked by brands, we decided to choose the visual dot probe task as an experimental paradigm. This procedure, one of the most commonly used in studies of attentional bias (Mogg et al., 2003; Yiend, 2010), allows to examine full time-course of the effect and obtain measures of all three basic components, i.e., orientation, maintenance, and disengagement (Fox et al., 2001; Mogg et al., 2003; Pool et al., 2016).

In the original dot probe task (MacLeod et al., 1986) a pair of stimuli appears, of which one is neutral, and the other is affectively valenced. After they disappear, a small dot replaces one of them, and the participant’s task is to react to the probe as quickly as possible. The behavioral form of this procedure relies on the idea that the distribution of attention can be assessed by investigating reaction times to the probes. The participants should react faster to probes appearing in the location of the attended stimulus. Depending on the stimulus exposure time, initial orienting (with durations < 500 ms) or maintenance (longer exposure times) of attention can be inferred (Mogg et al., 2003). Dot probe task provides even more direct insights into the time-course of attentional bias if used together with eye-tracking recordings (Field et al., 2009; Gao et al., 2011). Eye movements are an objective, directly observable indicator of visual attention (Hoffman and Subramaniam, 1995; Kowler et al., 1995; Moray, 2007). Furthermore, the anatomical overlap of brain areas responsible for oculomotor and attentional processes found on a neural level (Corbetta et al., 1998) makes the conclusions about the bias more theoretically valid. Additionally, eye-tracking version of the dot probe procedure provides superior reliability of measurement compared to the behavioral version of the task (Marks et al., 2014; Christiansen et al., 2015).

Interpretation of dot probe task results rests on the assumption that gaze behavior in each trial might be mapped onto three different aspects of attentional functioning: orienting, maintenance, and disengagement (Mogg et al., 2003). It is important to notice that each of these concepts has many accepted operationalizations. While designing our study we could not rely on the existing literature on the attentional bias evoked by brands, so we decided to use a wide range of indices maximizing the informative value of our analysis. Thus, attentional orienting was measured as the direction of the first saccade after the onset of the pair of pictures (Mogg et al., 2003, 2007; Field et al., 2004; Garner et al., 2006; Werthmann et al., 2011) and its latency (Mogg et al., 2003). Maintenance was examined by assessing the initial fixation duration (Mogg et al., 2003; Werthmann et al., 2011, 2013), initial gaze duration (Mogg et al., 2003; Werthmann et al., 2013), and total gaze duration (Mogg et al., 2003; Castellanos et al., 2009; Schofield et al., 2012; Arditte and Joormann, 2014), as well as proportion of total gaze time spent on the preferred brand picture and probability of attending preferred vs. non-preferred brand picture at least once during the course of the trial. The disengagement effects were measured as the time needed to start a saccade from currently attended picture toward a dot presented on the other side of the screen (Schofield et al., 2012). Some of the indices used in the dot probe literature, such as the number of shifts between the pictures (Field et al., 2004; Schofield et al., 2012), were not taken into account in our study, as we found them non-informative in the context of fairly limited picture exposition time (1000 ms).

To address the issue of specific attentional bias toward cigarette brands directly, we used a within-subject design in which participants performed the dot probe task with pairs of stimuli depicting packs of cigarettes solely – there were no photos representing any other objects. As cigarette packs are highly standardized, the branding information was the only factor influencing the gaze patterns. That allowed us to test our key hypothesis based on the premises presented above: we expected that photos presenting participants’ favorite brand would be processed preferentially, leading to the attentional bias.

The second goal of our study was to identify specific stages of the stimulus processing affected by the bias. We expected brand-preference to reveal itself in the later stages of the trial, but not in the initial saccade. This hypothesis was based on two premises, both leading to the similar predictions. Firstly, due to the high similarity of all the cigarette packs, we did not expect the brands to be easy to recognize. The center of the cigarette packs, which contained the brand logo in the majority of the pictures, was positioned 6.2° away from the fixation point. In this setting brand perception relied on the parafoveal vision which offers significantly lower visual acuity than central vision (Pelli and Tillman, 2008), thus making the identification process relatively difficult. Secondly, brand identification is a fairly complex process, even if extensively trained in the case of commonly used products. As shown in a study by Ellis et al. (2010) average reaction times in a brand recognition task might easily exceed 700 ms despite the optimal presentation and using material well-known to participants. In conclusion, we expected brand-related bias to emerge during maintenance and disengagement, but not in the (early) orienting phase of the trial determined by the direction of the initial saccade.

## Materials and Methods

### Participants

Participants were recruited by an online screening questionnaire which included questions concerning demographic variables, most frequently used cigarette brands, and strength of nicotine dependence (Heatherton et al., 1991). We invited to the lab 40 smokers using one of the three brands of cigarettes reported most frequently during online screening (Marlboro, L&M, LD) and with normal or corrected-to-normal vision. Three participants were excluded from analysis due to the poor quality of eye-tracking data. Thus, the effective sample size consisted of 37 subjects (20 females), aged 18 to 35 (*M* = 24.43, *SD* = 4.85). Mean score on the strength of nicotine dependence questionnaire was 5.57 (*SD* = 1.61). Scores over 5 are indicative of moderate, and over 6 – of strong nicotine dependence (Fagerström et al., 1990). In the final sample 20 participants declared L&M as their most frequently used brand, 15 reported Marlboro, and just two – LD.

### Stimuli

We used color pictures of cigarette packs in various positions made by a professional photographer especially for the purpose of the study. Three most popular brands declared by the participants were used: Marlboro, L&M, and LD. Additionally, one of the rarely reported brands (Winston) was used in the training phase and filler trials. Three varieties of each brand were photographed, differing in pack designs and colors (red, blue, and silver editions of the L&M and LD, and red, gold, and silver of Marlboro). All the packs were presented on a white background with constant lighting, exposure, and shooting distance parameters. Several viewing angles (frontal, rotated horizontally, and vertically) and pack arrangements (pack standing, lying, open, closed, etc.) were used for each brand and type of cigarettes. No additional objects were included in the pictures, although the cigarettes were visible inside the opened packs in some of the photos. Visual properties such as contrast and luminance were kept constant across picture sets, for all the common elements of the packages (i.e., health warnings). Mean luminance varied slightly across picture sets, mainly as a function of the color varieties used for each brand. Mean values for Marlboro, L&M, and LD were (respectively): 215.8 (*SD* = 7.51), 211.8 (*SD* = 3.61), 219.1 (*SD* = 3.91). All the photos are available on request from the authors.

### Procedure

The participants were asked to smoke their last cigarette 1 h before arriving at the laboratory. After arrival, the participants signed the written informed consent form and answered the following question concerning their current cigarette craving: on the scale below, please rate your urge to smoke in the present moment. A seven-point Likert scale was used, with “1” labeled “I do not feel like smoking at all” and “7” labeled “I would like to smoke very much right now.” They were seated in front of a 1920x1080 Benq XL2420T monitor, at a 70 cm distance, with the chin and forehead supported by a chinrest. Psychophysiological reactions were also recorded but are not the focus of this paper. The participants completed a dot probe procedure and several other tasks (not reported here), however, the dot probe was always performed at the beginning of the session. Before leaving, they reported their cigarette craving again by responding to the same question as at the beginning and filled out a questionnaire concerning cigarette brand attitudes. Afterward, they were debriefed and paid 30PLN (approximately 7 Euro).

The study design and the informed consent form were approved by the Ethics Committee of the SWPS University of Social Sciences and Humanities. The participants were informed that their data will remain anonymous and will be used only for the purpose of statistical analyses and that they will receive the monetary gratification regardless of whether they complete the whole procedure or quit at any time.

### Dot Probe Task

The dot probe task was adapted from Lipp and Derakshan (2005) and programmed in ePrime 2.0 (Psychology Software Tools, Sharpsburg, PA, United States). It included 100 pictures of 4 cigarette brands (30 pictures of each of the three target brands and 10 filler pictures).

**FIGURE 1:**
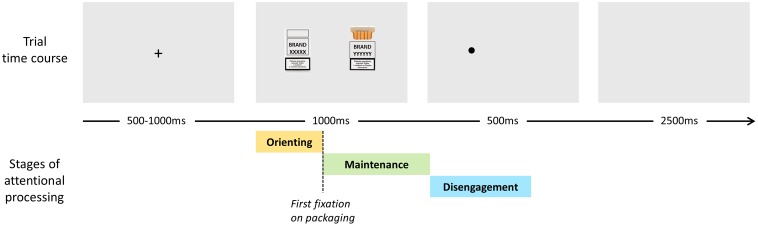
Time course of a dot probe trial and stages of attentional processing.

Eye movements were recorded by an infrared Eyelink 1000 (SR Research, Ottawa, ON, Canada) eye tracker from the left eye with 1000 Hz frequency. Calibration was performed before the start of the procedure, followed by validation. The participants were instructed to explore the pictures freely, and then move their gaze to the dot as quickly as possible after its appearance. The task started with eight training trials with filler pictures as stimuli. The main procedure consisted of 15 blocks of 12 trials each. At the beginning of each block a drift correction was performed, after which one trial with filler pictures (not included in the analyses) was displayed. Each trial started with a fixation cross displayed for 500–1000 ms (varying randomly by 125 ms from trial to trial), after which a pair of pictures was shown for 1000 ms, followed immediately by a dot replacing one of the pictures, exposed for 500 ms. After another 2500 ms, the next trial started (**Figure 1**). In each trial, a pair of pictures showing packs of two different cigarette brands was displayed. Each brand appeared with equal frequency, so two-thirds of the trials contained a photo depicting the packaging of the cigarette brand smoked regularly by the subject. The pictures were shown symmetrically to the left and right of the center of the screen. The size of each photo including the background was 500 × 500 px, and the distance between their inner edges was 130 px (3°). Each photograph was shown twice on the left, and twice on the right side of the screen during the whole procedure.

### Brand Attitude Questionnaire

This questionnaire included eight questions concerning the attitude toward each of the three brands. Every participant responded to all the questions referring to the three brands. The response format was Likert scale (1–7). The following questions were asked: *I think these are the best cigarettes; I always buy cigarettes of this brand; The cigarettes of this brand have the best flavor; I like to smoke them; Cigarettes of this brand are of a high quality; The price of these cigarettes is advantageous; I have positive associations with this cigarette brand; I like the packaging of these cigarettes.* Cronbach’s alpha coefficients for the Marlboro, LM, and L&D brands were 0.88, 0.95, and 0.94, respectively.

### Data Filtering and Aggregation

For each subject, the preferred brand was determined using data collected during the screening phase of the study. It was defined as the one declared to be most frequently used. Thus, for every participant, the stimuli were classified as either representing preferred (“own”) or other two (non-preferred) brands. High correlation between the reported frequency of use and brand preference did not allow us to compare participants with coherent vs. incoherent declarations systematically. It was possible, however, to repeat the analysis on a subgroup of 32 participants (86.5% of the final sample) whose preference ratings matched declared behavior. Obtained results closely replicated those obtained in the whole sample.

All analyses were based on data obtained in trials where one of the presented photos depicted packaging of participants’ preferred brand, that is 120 trials. Analysis of disengagement indicators was further limited to trials in which the dot appeared on the opposite side of the screen relatively to the currently fixated picture. The average expected number of such trials was 60 per participant, however, as gaze and picture positions were unpredictable, it varied significantly across subjects.

Prior to aggregation, single trial data were filtered. In the analysis of the early-stage bias, trials were considered valid if (1) participants were fixated at the center of the screen when pictures appeared, (2) first saccade was directed toward one of the pictures, (3) there were no blinks during the first saccade, (4) direction of the saccade could be identified, and (5) saccade latency was between 100 and 1000 ms from the pictures’ onset (Mogg et al., 2003). As a consequence, first saccade analysis encompassed 77.8% of the initial number of trials. For indices describing gaze behavior during picture presentation, we used an additional filter. Gaze duration data were considered valid if the values exceeded 100 ms, leaving 72.4% of the first fixations trials, 74.4% of the first gaze trials and 77.3% total gaze duration trials.

Disengagement latency was defined as an interval between the appearance of the dot and the beginning of the first saccade toward it. Trials were included in this analysis only if (1) participants performed a saccade starting from one of the photos, (2) the saccade was directed toward the dot, (3) its latency was above 50 and below 750 ms (from the dot onset), and (4) it did not contain blinks. We decided to use lower cut-off values in dot probe disengagement trial filtering, as the time course of this part of each trial was fully predictable, and participants were motivated by the instruction to attend to the dot as fast as possible. In these specific conditions the saccade preparation process could start long before the appearance of the dot. Due to the fluctuations in the number of available trials (dot position was unpredictable), other filtering criteria, participants’ strong preference to dwell on the photo of the preferred brand, there was a large disproportion in the number of valid trials between the two conditions. Significantly more saccades started from the picture of the preferred brand packaging than from the pictures of the other brands. Therefore, the disengagement analysis was performed after removing data from three subjects who provided less than eight valid data points in any of the conditions. Of the initial number of trials, 64.0% were available for analysis.

All the latencies and fixation durations are expressed in milliseconds. Initial saccade bias is represented as a proportion (the number of first saccades toward the preferred brand divided by the number of valid trials). Therefore, a score of 0.50 indicates no effect, and higher values indicate a bias toward the preferred brand. The proportion of fixation time was computed as the sum of gaze durations on the preferred brand packaging divided by the total gaze duration in a given trial, so, again, scores above 0.50 were indicative of a bias toward the preferred brand.

Average scores for all latency and duration indices were computed after removing outliers within each subject and condition defined as values deviating more than three standard deviations from the mean scores. The proportion of removed observations did not exceeded 1.6% of trials for any of the indices.

## Results

Two-sided paired-samples *t*-tests were used to test equality of means in the compared conditions (processing of the preferred vs. non-preferred brand pictures). Hypotheses concerning bias scores were tested using one-sample *t*-test with 0.50 reference value. Additionally, Bayes Factors for all comparisons were estimated using Bayesian *t*-tests (Rouder et al., 2009) with a default Cauchy prior (width of *r* = 0.707). The results of Bayesian *t*-test are reported as a proportion of likelihoods of data under the alternative hypothesis compared to likelihood under the null (BF_10_). Statistical analyses were performed using R (R Core Team, 2016) and JASP (JASP Team, 2017). All the *p*-values are uncorrected, however, applying false discovery rate procedure (Benjamini and Hochberg, 1995) did not modify any of the decisions concerning the significance of the reported *t*-tests.

Initial saccade direction and first saccade latency were used to assess early-stage processing of brand images. The average proportion of saccades toward the preferred brand was 0.524 (*SD* = 0.087) and showed no significant effect, *t*(36) = 1.70, *p* = 0.097, *d* = 0.28, BF_10_ = 0.66. Similarly, no differences between the mean latency of first saccades toward the preferred vs. non-preferred brand were detected, *t*(36) = 1.62, *p* = 0.115, *d* = -0.26, BF_10_ = 0.58 (see **Table 1** for means and standard deviations for this and all the following comparisons).

**Table 1:** Mean scores and standard deviations of indices quantifying eye-movements.

		Own brand		Other brands	
Eye-movements indices	*N*	Mean	*SD*	Mean	*SD*
First saccade latency (ms)	37	347	56	341	57
First fixation duration (ms)^+^	37	247	66	229	72
First gaze duration (ms)^∗∗∗^	37	410	75	326	92
Total gaze duration during picture exposure (ms)^∗∗∗^	37	312	65	214	70
Probability of fixating on packaging at least once^∗∗∗^	37	0.862	0.114	0.720	0.178
Latency of saccade toward the dot (ms)^∗∗^	34	329	60	288	71

^+^*p = 0.52, *^∗∗^p* < 0.01, *^∗∗∗^p* < 0.001*.

Five indices quantified eye movements that followed the initial saccade and took place during the part of the trial when pictures were presented. Duration of the first fixation was the only one where the differences related to brand preference were only marginally significant and provided no conclusive information as indicated by the Bayes factor value close to 1, *t*(36) = 2.01, *p* = 0.052, *d* = 0.33, BF_10_ = 1.07. All the remaining indices revealed a robust bias toward participants’ preferred brand, which manifested as longer average duration of first gaze [*t*(36) = 5.42, *p* < 0.001, *d* = 0.89, BF_10_ = 4637.97], total gaze time [*t*(36) = 4.70, *p* < 0.001, *d* = 0.77, BF_10_ = 604.59], as well as the proportion of gaze time spent on the preferred brand exceeding significantly the reference value of 0.50 [*M* = 0.589, *SD* = 0.118, *t*(36) = 4.56, *p* < 0.001, *d* = 0.75, BF_10_ = 414.19]. Furthermore, the probability that participants will attend the photo of the preferred brand packaging at least once during the trial was significantly higher for the preferred than for the remaining brands, *t*(36) = 4.33, *p* < 0.001, *d* = 0.71, BF_10_ = 221.00. Thus, participants fixating at the beginning of the trial at the picture of their preferred brand shifted their attention away from it less often than when the first attended picture depicted packaging of another cigarette brand.

The last analysis concerned average latencies of the first saccades toward the dot which were used as a measure of disengagement difficulty. In line with the effects reported above, average latencies were significantly shorter if participants were looking at the packaging of non-preferred brands before the dot appeared, *t*(33) = 3.43, *p* = 0.002, *d* = 0.588, BF_10_ = 20.75.

Additionally, for the same set of eight indices we repeated the analyses within subgroups of L&M and Marlboro users (it was impossible to run such an analysis for LD smokers, due to the limited group size). The overall pattern of results in both cases closely replicated the effects obtained for all participants (for details see **Table 2**). Still, when compared with the results reported for the complete sample, two important differences are worth noticing. Firstly, in the Marlboro group, the initial orienting effect was statistically significant (however the Bayes factor value suggested only a weak evidence against the null hypothesis). Secondly, in the L&M group, there was no detectable effect on the latency of the saccade toward the dot (disengagement index).

**Table 2:** Eye movement biases toward “own” vs. “other” brands for participants declaring Marlboro or L&M as the most commonly used brands.

	Marlboro				L&M			
Eye-movements indices	*N*	*t*	*d*	*BF*	*N*	*t*	*d*	*BF*
Proportion of saccades toward the preferred brand	15	2.34^∗^	0.60	2.03	20	-0.35	-0.08	0.25
First saccade latency	15	-1.89	-0.49	1.07	20	-0.10	0.02	0.23
First fixation duration	15	2.94^∗^	0.76	5.27	20	-0.58	-0.13	0.27
First gaze duration	15	4.54^∗∗∗^	1.17	75.71	20	3.03^∗∗^	0.68	6.99
Total gaze duration during picture exposure	15	4.32^∗∗∗^	1.12	52.57	20	2.35^∗^	0.53	2.12
Proportion of gaze time spent on the preferred brand	15	4.20^∗∗∗^	1.08	42.72	20	2.31^∗^	0.52	1.96
Probability of fixating on packaging at least once	15	3.72^∗∗^	0.96	19.09	20	2.63^∗^	0.59	3.39
Latency of saccade toward the dot	12	3.39^∗∗^	0.98	8.98	20	1.72	0.39	0.81

^∗^*p < 0.05, ^∗∗^*p* < 0.01, ^∗∗∗^*p* < 0.001; BF, Bayes factor*.

The second set of analyses focused on the strength of relationships between eye-movement measures and cigarette craving intensity as well as addiction strength. None of these correlations were significant (**Table 3**).

**Table 3:** Correlations between eye-movement indices, cigarette craving, addiction strength, and attitude measures.

		Cigarette craving		Addiction strength “own” brand		Attitude toward score		Attitude difference	
Eye-movements indices	*N*	*r*	*BF*	*r*	*BF*	*r*	*BF*	*r*	*BF*
Proportion of saccades toward the preferred brand	37	0.288	0.86	0.140	0.28	0.244	0.57	0.092	0.24
First saccade latency	37	0.014	0.21	0.165	0.32	-0.163	0.32	-0.244	0.57
First fixation duration	37	0.075	0.23	-0.102	0.24	0.308	1.07	0.406^∗^	4.05
First gaze duration	37	0.152	0.30	-0.026	0.21	0.353^∗^	1.87	0.331^∗^	1.42
Total gaze duration during picture exposure	37	0.156	0.31	0.039	0.21	0.408^∗^	4.18	0.337^∗^	1.52
Proportion of gaze time spent on the preferred brand	37	0.157	0.31	0.049	0.21	0.392^∗^	3.28	0.305	1.03
Probability of fixating on packaging at least once	37	0.176	0.35	0.060	0.22	0.316	1.17	0.194	0.39
Latency of saccade toward the dot	34	-0.126	0.27	0.246	0.55	0.028	0.22	0.053	0.22

^∗^*p < 0.05; Pearson’s *r*; BF, Bayes factor; Attitude difference score is the mean of the attitudes toward the two “other” brands subtracted from the attitude toward“own” brand*.

Finally, we analyzed declared attitudes toward brands. Following our expectations, participants evaluated “own” brand (*M* = 5.63, *SD* = 1.09) significantly higher than the two non-preferred brands averaged (*M* = 3.27, *SD* = 0.93), *t*(36) = 9.51, *p* < 0.001, *d* = 1.56, BF_10_ > 1000. At the same time, attitude toward the most frequently used brand was positively correlated with three gaze duration measures capturing participants’ behavior during the maintenance phase of the trial: first gaze and total gaze duration, as well as the proportion of the gaze time spent on the preferred brand (for details see **Table 3**). We repeated this analysis for an additional measure of brand preference – attitude difference score computed as a difference in attitude score between the “own” and the remaining two “other” brands. Again, three significant positive correlations were found linking the relative preference of the “own” brand with durations of the first fixation, first gaze, and total gaze duration (**Table 3**).

## Discussion

Our study focused on detecting an attentional bias toward preferred cigarette brand among smokers. Obtained results confirmed our initial expectations based on theoretical premises: the dot probe task allows to detect a systematic bias toward “own” brand of cigarettes. In particular, reliable effects were observed in later stages of attentional processing, namely maintenance and disengagement, whereas no significant differences were found in the initial orienting response. Since we are not aware of any other studies investigating the attentional biases toward brands, we will interpret our results in the context of existing research on affective and reward-related stimuli.

Pictorial representations of any addictive substance seem to be a special category which is both personally relevant and associated with a reward for the regular users, hence we might expect it to evoke attentional bias (Purkis et al., 2011). Our results show that the participants exhibited attentional biases toward their favorite brand, even though all the presented stimuli were related to smoking. This pattern of results suggests that branding information might be an important moderator amplifying the bias. In the light of the incentive-salience model, we could say that the regularly used brand has higher incentive salience and, therefore, stronger “attention-grabbing” properties (Robinson and Berridge, 2000).

Importantly, significant effects of brands were only visible in the later stages of attentional processing, i.e., in the maintenance and disengagement of attention. These results are in contrast to the recent metaanalysis (Pool et al., 2016) which showed that the attentional bias for positive emotional stimuli was larger for measures of initial orienting than for disengagement which is a later component of attentional processing. It is important to notice, however, that in the majority of studies included in this metaanalysis stimuli were fairly easy to discriminate – they either depicted visually distinct categories of objects (e.g., Tapper et al., 2010) or facial expressions (Isaacowitz et al., 2006; Joormann and Gotlib, 2007) which are known to be processed in the specific and extremely efficient way (Kesler-West et al., 2001; Hariri et al., 2002; Britton et al., 2006).

In the case of our study, it was not clear whether more complex stimuli, i.e., branded cigarette packs, would also evoke attentional biases during all the stages of processing. As we wanted to avoid perceptual habituation and introduced a wide range of packaging positions, angles, etc. the brand logos and other characteristic features were not always presented in optimal viewing positions which further increased the difficulty of brand processing. In our opinion, it is unlikely that they could be processed quickly enough to evoke differences in early orienting reaction. It is even less likely concerning relatively large visual angle between the presented pictures, the fully randomized pairing of stimuli, and a large number of unique photos of each of the brands. Our study has no direct analogs in literature, however this interpretation might be partially supported by related results. For example, Bradley et al. (2004) found that briefly presented, masked smoking-related stimuli did not evoke attentional bias and suggested that conscious identification might be necessary to evoke this effect. In our study, where the key manipulated factor was particular brand identity appearing in the context of high interference, Bradley and colleagues’ conclusion might be applicable as well.

We should also keep in mind that the low-level visual features of the stimuli were not fully controlled in our study. The photo sets created for each of the brands differed slightly in luminance, and some further differences in visual saliency were introduced by color variations, characteristics of packaging design, etc. Saliency is known to significantly impact both early deployment of attention (Nardo et al., 2011, 2014; Borji et al., 2015) and later stages of stimulus processing (Foulsham and Underwood, 2008; Santangelo, 2015), so it might be treated as a potential confounding factor in our design. Nevertheless, the results of the follow-up analyses conducted within subgroups of L&M and Marlboro users (**Table 2**) strongly suggest that differences in low-level characteristics of the photos cannot fully explain the bias effects in our study. Importantly, it does not preclude the conclusion that saliency is a significant moderating factor intensifying the brand related bias. That possibility is certainly worth systematic investigation in further research.

Similarly, our design did not allow to assess the relative contribution of familiarity to the emergence of the bias. Participants might have preferred the brand *per se*, or just prefer “own” brand logo as a more familiar object. More precise identification of the causal mechanisms involved in the emergence of the brand-related bias is both theoretically interesting and experimentally challenging. Usage and brand preference are strongly correlated with familiarity. Brand logo exposure might, in turn, contribute to the more positive assessment evoked, for example, by mere exposure (Olson and Thjomoe, 2003) or higher perceptual fluency (Lee and Labroo, 2004). Unfortunately, none of these variables is easy to manipulate experimentally. Hence, disentangling the relative contribution of brand preference and familiarity in understanding bias effects remains a task of the future.

Our paper describes a previously unreported effect and its results certainly require replication. At the same time, however, it also clearly indicates the need to develop theoretical models of attentional bias encompassing branding information as the additional dimension of stimulus saliency. One of the most important problems that arise in this setting is the need to better understand the interplay between the addiction- and brand-related biases. Regrettably, our study does not allow to resolve this issue, as all the stimuli were directly related to participants’ addiction, and it was impossible to compare them against the neutral photos not related to smoking.

Our results might also have important implications for studies using attentional bias measures as tools supporting the diagnosis or monitoring treatment-induced changes (Streeter et al., 2008). The observed effects sizes suggest that, at least in the context of nicotine addiction, matching the stimuli with individual brand preferences might significantly affect participants’ behavior and, in consequence, the properties of the obtained attentional bias estimates. From the psychometric perspective, choosing the right stimuli might affect construct validity of the measures, by making the reactions more relevant to real addiction-related behavior, and consequently leading to superior reliability and improved external validity. These claims gain initial support from the results of the correlational analyses (reported in **Table 3**), as proposed indices capturing the brand-related bias correlate positively with two measures of attitudes toward the preferred cigarette brands.

Despite promising results, we are well aware of some significant limitations of our study. Firstly, it did not allow to separate the effects of actual brand preference expressed in regular use, from brand preference defined as a positive attitude or superior brand image. Considering the habitual nature of the addiction, we expect that the bias is mainly driven by gradually developing associations between the most commonly used brand and reinforcements provided by smoking. This claim, although theoretically justified by the incentive-salience model, requires direct empirical testing. Secondly, in our study design, we decided not to manipulate the time course of the trial. As a consequence, it was also impossible to disentangle two theoretically distinct (but related) aspects of the bias: the time dynamics (determined by the presentation times of the pictures, latency of the dot appearance, etc.) and the scope of the bias, which in our results seems to be limited to the maintenance and disengagement. Thirdly, future replications of our study could include some additional moderators, such as manipulation of deprivation or control of the addiction strength. In our study follow-up analysis showed that neither of these variables was significantly related to the bias measures. However, it could be explained by the fact that in both cases the variance of potential moderators was limited. Our sample was homogenous in terms of the addiction strength – only three participants had results indicating moderate or low addiction (3 or fewer points in Fagerström scale, Heatherton et al., 1991) and differences in craving levels were diminished – all participants were asked to smoke their last cigarette 1 h before arriving at the laboratory.

The last group of questions that might be raised in the context of our study is, in our opinion, the most theoretically interesting. Given the strong addictive properties of nicotine (Nutt et al., 2007) and postulated incentive-related mechanism responsible for the emergence of the bias, the generalizability of the brand related effects becomes crucial. Based on our study, we cannot conclude to what extent the observed bias is limited by some specific aspects of the population of smokers. Can we expect to observe similar biases toward brands of products containing other – potentially addictive – substances? Is this effect moderated by the frequency of contact with a product (for example alcohol is drunk less often than cigarettes are smoked)? Could similar effects hold among regular and loyal users who are not addicted? Broadening the scope of study even further: is it possible to measure brand bias among users of products which are rewarding, but not addictive or brands which are highly emotionally involving, but have only symbolic meaning (sport team logos or national flags)?

In sum, our study provides initial evidence that attentional bias effects in nicotine addiction are brand-specific and might be observed in both maintenance and disengagement stages of stimulus processing. We believe that the results of the presented study provide a promising starting point for future research efforts. The examination of attentional biases toward brands might contribute to better understanding of the mechanisms involved in the formation and maintenance of attentional biases, and therefore have important implications for both marketing and clinical research. From a measurement-oriented perspective, it might also help us operationalize important aspects of attitudes and preferences more accurately, using innovative, non-declarative measures.

## Ethics Statement

This study was carried out in accordance with the recommendations of the Ethics Committee of SWPS University of Social Sciences and Humanities with written informed consent from all subjects. All subjects gave written informed consent in accordance with the Declaration of Helsinki. The protocol was approved by the Ethics Committee of SWPS University of Social Sciences and Humanities in Warsaw.

## Author Contributions

MB and ED contributed to the study design. Data collection was performed under the supervision of ED. MB performed data analysis. Both authors wrote the paper and approved the final version of the manuscript. Authors declare equal contribution to the work.

## Conflict of Interest Statement

The authors declare that the research was conducted in the absence of any commercial or financial relationships that could be construed as a potential conflict of interest.
